# Working mechanisms of the use and acceptability of ecological momentary interventions: a realist evaluation of a guided self-help ecological momentary intervention targeting self-esteem

**DOI:** 10.1186/s12889-024-19143-z

**Published:** 2024-06-19

**Authors:** Mary Rose Postma, Suzanne Vrancken, Maud Daemen, Iris Hoes-van der Meulen, Nele Volbragt, Philippe Delespaul, Lieuwe de Haan, Marieke van der Pluijm, Josefien Johanna Froukje Breedvelt, Mark van der Gaag, Ramon Lindauer, David van den Berg, Claudi Bockting, Therese van Amelsvoort, Matthias Schwannauer, Lawrence Doi, Ulrich Reininghaus

**Affiliations:** 1 Department of Psychiatry and Neuropychology, School for Mental Health and Neuroscience, Maastricht University, Vijverdalseweg 1, Maastricht, 6226 NB the Netherlands; 2 Mondriaan Mental Health Center, Heerlen, The Netherlands; 3 Prodeba Mental Health Center, Leiden, The Netherlands; 4 Department of Psychiatry, Amsterdam Public Health, Amsterdam University Medical Centers (location AMC), Amsterdam, The Netherlands; 5 Department of Child and Adolescent Psychiatry, Institute of Psychiatry, Psychology and Neuroscience, King’s College London, London, London, UK; 6 Centre for Urban Mental Health, University of Amsterdam, Amsterdam, The Netherlands; 7 Department of Clinical Psychology, VU University, Amsterdam, The Netherlands; 8 Department of Child and Adolescent Psychiatry, Amsterdam UMC, location Academic Medical Center, Amsterdam, The Netherlands; 9 Levvel, Academic Centre for Child and Adolescent Psychiatry, Amsterdam, The Netherlands; 10 Parnassia Academy, The Hague, The Netherlands; 11 School of Health in Social Science, University of Edinburgh, Edinburgh, UK; 12 Department of Public Mental Health, Medical Faculty Mannheim, Central Institute of Mental Health, Heidelberg University, Mannheim, Germany; 13 ESRC Centre for Society and Mental Health and Social Epidemiology Research Group, King’s College London, London, UK; 14 Health Service and Population Research Department, Centre for Epidemiology and Public Health, Institute of Psychiatry, Psychology & Neuroscience, King’s College London, London, UK

**Keywords:** Ecological momentary intervention, Self-esteem, Target mechanism, Working mechanism

## Abstract

**Background:**

Technology improves accessibility of psychological interventions for youth. An ecological momentary intervention (EMI) is a digital intervention geared toward intervening in daily life to enhance the generalizability and ecological validity, and to be able to intervene in moments most needed. Identifying working mechanisms of the use of ecological momentary interventions might generate insights to improve interventions.

**Methods:**

The present study investigates the working mechanisms of the use and acceptability of an ecological momentary intervention, named SELFIE, targeting self-esteem in youth exposed to childhood trauma, and evaluates under what circumstances these mechanisms of use and acceptability do or do not come into play. A realist evaluation approach was used for developing initial program theories (data: expert interviews and a stakeholders focus group), and subsequently testing (data: 15 interviews with participants, a focus group with therapists, debriefing questionnaire), and refining them.

**Results:**

The SELFIE intervention is offered through a smartphone application enabling constant availability of the intervention and thereby increasing accessibility and feasibility. When the intervention was offered on their personal smartphone, this enhanced a sense of privacy and less hesitance in engaging with the app, leading to increased disclosure and active participation. Further, the smartphone application facilitates the practice of skills in daily life, supporting the repeated practice of exercises in different situations leading to the generalizability of the effect. Buffering against technical malfunction seemed important to decrease its possible negative effects.

**Conclusions:**

This study enhanced our understanding of possible working mechanisms in EMIs, such as the constant availability supporting increased accessibility and feasibility, for which the use of the personal smartphone was experienced as a facilitating context. Hereby, the current study contributes to relatively limited research in this field. For the field to move forward, mechanisms of use, and acceptability of EMIs need to be understood. It is strongly recommended that alongside efficacy trials of an EMI on specific target mechanisms, a process evaluation is conducted investigating the working mechanisms of use.

**Trial registration:**

The current paper reports on a realist evaluation within the SELFIE trial (Netherlands Trial Register NL7129 (NTR7475)).

**Supplementary Information:**

The online version contains supplementary material available at 10.1186/s12889-024-19143-z.

## Background

Youth, referring to a period of transitioning from childhood to adulthood spanning from 12 to 25 years of age, is a crucial time regarding the development of psychosocial capacities and the onset of mental disorders [1]. Specifically, 62.5% of individuals report an onset of a mental disorder before the age of 25 [2]. Thus, it is important to intervene in youth, however, a gap exists between their needs and modus operandi, and available care [3–6]. The importance of overcoming this gap has extensively been underscored in clinical work as well as research and has led to efforts for international reform of youth mental health [7]. In recent years, mHealth (i.e. mobile health, interventions making use of mobile devices) increasingly provides solutions to bridge this gap. Technology can make psychological interventions more accessible for youth, who are ‘digital natives’ [8]. An abundance of apps that promise to relieve psychological stress and enhance mental well-being are available in major app stores. But only a small number of applications are tested for efficacy [9] and most are not grounded in sound psychological theories, such as cognitive behavioral interventions [10].

Ecological momentary interventions (EMI) are typically digital interventions [11, 12]. However, the development of EMIs was not triggered by the digital transformation, it is rooted in ecological psychology, for which digital advances (e.g. with smartphones) provide new assessment methods and treatment delivery channels. Ecological psychology aims to study experience and behavior in the context of normal daily life. Therefore it uses Ecological Momentary Assessment (EMA) [11]. EMIs take this a step further and assume that experience and behavior are not only situated in, but also most amenable to change in momentary daily life situations [13]. EMIs are designed to intervene in daily life, herewith enhancing the generalizability and ecological validity of the learning situation, and focus interventions to target moments [14]. Moreover, EMIs can prompt behaviour, experiences, and assignments in real life, independant of contact with a therapist.

EMIs were developed for different mental health problems such as mood [15, 16], anxiety [17, 18], and substance use disorders [19–21]. In addition, EMIs can focus on transdiagnostic intervention elements [22, 23]. Unfortunately, still a limited number of studies rigorously research the effect of EMIs and several major (transdiagnostic) psychological domains remain unaddressed [24–26].

The term ‘just-in-time adaptive intervention’ (JITAI) has also been used in literature to describe an intervention delivered in daily life that is customized to an individual’s state aiming to provide the right type of support varying over time, using information assessed through e.g. EMA [27]. Even though there are similarities between EMI and JITAI (i.e. intervening in the moment when needed and prompted), JITAI emphasizes the specific element of adaptation over time (not inherent to EMI perse) and, hence, may be termed as a subclass of EMI [24]. However, from the outset, EMIs used adaptive strategies, e.g., referred to as interactive tasks in recent studies [28, 29], and as such adaptation of the intervention is inherent to EMIs. For this paper, with the EMI under study delivering interactive tasks, the term EMI will be used.

### EMI research

To enhance the field of EMI research an important challenge is the need for high-quality trials [25, 30, 31]. The SELFIE trial targeting self-esteem, and the EMI compass trial targeting resilience through a compassion-focused intervention, are particular examples for targeting mechanisms that may confer the development of mental disorders at a later age [23, 29, 31]. Research moves from knowing that psychological interventions work to understanding how they work [32]. This requires understanding the ‘mechanism’, defined as the roots of the effect, i.e., the processes of events that are responsible for the change; the reasons why change occurred or how change came about” [33]. Thus, e.g. self-esteem may be coined as an ‘active ingredient’ for psychological interventions [34–36], and could be targeted as a mechanism of influence in the development and maintenance of psychological disorders [37]. Such a mechanism is hereafter referred to as ‘target mechanism’. Specifically in EMI research, the feasibility and effectiveness of intervening in target mechanisms in daily life (a core element of EMI) is researched. The testing of ecological interventionist causal models [12], offers an addition to existing research in testing whether an EMI can modify a target mechanism in daily life, and secondly test whether producing changes in the mechanism in daily life leads to changes in intended psychological outcomes. Nonetheless, how the target mechanism is being influenced remains unanswered. This influencing of the target mechanism may be seen as a mechanism in itself, defined as an explanatory theory of what elements of (the mode of delivery of) an intervention causes changes in individual behavior relating to the target mechanism [38], and will hereafter be referred to as ‘working mechanism’. Current research should pay attention to the target mechanisms as well as the working mechanisms of the intervention itself to improve the use of EMIs in clinical care [11].

Various working mechanisms have been pointed out in previous work. For example, EMIs are available at any given time and in every given context. Individuals may feel more equipped to apply new behaviours and skills in their actual experience in real life with the extra support of an EMI [14, 39]. Additionally, based on assessment responses, EMIs can tailor (the content of) the intervention to specific needs at specific times, based on, for example, need of additional support, motivation or readiness to change [14]. EMIs can also prompt contextual reminders to trigger specific behaviours in specific contexts [24, 40, 41]. Another established working mechanism is self-monitoring, individuals can track their behaviours and become more aware of them, which is necessary for change [41, 42]. In some EMIs individuals can connect with peers or friends for social support [14, 41]. Furthermore, personalized feedback on app progress can enhance self-awareness and motivation [15, 24, 40, 42], and goal-setting can increase motivation [14, 40]. Further, research has shown that guided self-help and face-to-face treatments may have comparable effects [43], and specifically for EMIs, guidance was proposed as an important working mechanism [10, 44]. Yet, limited research is available on how and for whom this mechanism works. Knowledge of working mechanisms is an important aspect to take into account when further implementing EMIs.

To understand the working mechanisms of an EMI, a realist evaluation method is a fitting approach because it develops a theory on how a program works, for whom, and under what circumstances [45]. Therefore, applying realist evaluation methodology within a EMI trial helps to advance current research. It also provides information to customize interventions [30]. Gaining insights into how and why an intervention works, would support future development and implementation of EMIs.

### SELFIE intervention

The current paper reports on a realist evaluation within the SELFIE trial (Netherlands Trial Register NL7129 (NTR7475)) of which details are available elsewhere [29, 31]. The SELFIE trial aimed to investigate the efficacy and clinical feasibility of SELFIE, which is a smartphone-based guided self-help intervention for improving self-esteem in youth exposed to childhood trauma, in a multi-center, parallel-group, assessor-blind randomized controlled trial (RCT). Their findings show improvement in the primary outcome of self-esteem at postpost-intervention and 6-month follow-up, and small to moderate effect sizes point towards beneficial effects on some secondary outcomes such as general psychopathology and quality of life [31]. The EMI under study in the SELFIE trial aimed to enhance self-esteem in youth (12–25 years) who have experienced childhood adversity (i.e., abuse, neglect, bullying, and/or household discord). A 6-week manualized intervention is delivered by trained SELFIE therapists, consisting of three face-to-face sessions, three standardized e-mail contacts, and an EMI administered through a smartphone-based app (i.e., the PsyMate® app), supporting the adaptive real-time and real-world transfer of intervention components tailored to moment, person, and context. By providing an ecologically valid, accessible, and personalized intervention, SELFIE aimed to tailor interventions to the needs of youth [11, 12, 14]. The preventive content, principles and techniques of the transdiagnostic SELFIE intervention have been based on the CBT model and interventions [46] and the self-help manual by de Neef [47].

### Aims

The aim of the present study was to investigate, within the SELFIE intervention, mechanisms of EMI and under what circumstances these mechanisms do or do not come into play.

## Methods

### Study design

A realist evaluation methodology was adopted to gain insight into EMI working mechanisms and consisted of three phases: (1) developing an initial programme theory (IPT), based on literature and two expert interviews and one focus group with stakeholders; (2) testing the IPT by comparing and explaining data offered by fifteen semi-structured interviews with youth who had received the SELFIE intervention, one focus-group with SELFIE therapists, and a debriefing questionnaire (*n* = 61); (3) refining the IPT. The guidelines offered by the RAMESES II reporting standards for realist evaluation [48] were followed, thereby adhering to the Standards for Reporting Qualitative Research (SRQR) [49].

Researchers undertaking this realist evaluation also partly played a role in developing (MP) and delivering the SELFIE intervention (MP, KS, SV). To minimize interpretation bias and influences due to a therapeutic relationship after delivering the intervention, researchers did not interview participants to whom they had previously delivered the SELFIE intervention. Neutrality in the process of data collection and analysis was ensured by reflexivity, i.e. documenting the progress, decisions, and motives of the researchers, aimed at supporting self-awareness concerning their role and impact on the research environment. In addition, frequent meetings with independent researchers (LD, MS, and a researcher conducting a realist evaluation in a different research trial) were held to discuss the researchers’ role.

The current realist evaluation was set in the context of the SELFIE trial [23, 29], and follows a previously undertaken realist evaluation of the SELFIE intervention [50]. This previously undertaken realist evaluation was a result of the known relevance of interventions aimed at low self-esteem in youth, and due to the complex nature of self-esteem and its targeting by an intervention, it was considered important to focus the analysis of the available qualitative data solely on characteristics and delivery of self-esteem interventions. The present realist evaluation analyses the same qualitative data, however, the analysis is aimed to investigate mechanisms of EMI, and under what circumstances these mechanisms do or do not come into play. The methods of the present realist evaluation, therefore, resemble the methods of the previous realist evaluation within the SELFIE trial but focus on the distinct aspect of EMI within the SELFIE intervention. A detailed description of the methods is shown in Table 1.

**Table 1:** Methods of recruitment, data collection, and analyses for phases 1 and 2

***Phase 1 (developing the IPTs)***	
*Stakeholders*	Reviewing literature supporting the development of the SELFIE trial and its intervention, informed the process of informant selection and the development of topic guides for interviews. Following the argument that the SELFIE intervention encompasses ‘self-esteem’ and ‘EMI’ as two key constructs, one expert (who was not involved in the development of the SELFIE intervention) for each construct was invited for individual expert interviews, aimed at identifying outcomes and key mechanisms. Furthermore, a focus group was organized with the stakeholders involved in developing and implementing the SELFIE intervention, aimed at making explicit the assumptions of the program developers and informing on relevant contexts. By combining expert interviews (not directly involved) and the stakeholder’s focus group (directly involved), it was possible to gain a range of perspectives on the key constructs of the SELFIE intervention.
*Recruitment and data collection*	For the individual expert interviews, two experts were informed about the SELFIE trial and the current realist evaluation and were invited to take part in an online video interview. Both experts agreed to participate. The experts were interviewed by SV and MP jointly with the use of a literature-informed topic guide (see Supplementary Materials 1 and 2 for the topic guides). A first analysis of the expert interviews aided in further developing a topic guide for the stakeholder’s focus group (see Supplementary Material 3). For this focus group, those who had been involved in the development of the SELFIE intervention were invited to join an online video focus group, and a date was planned on which all participants were available. The language used in this focus group was English (the non-native English-speaking stakeholders were familiar with the English language for meetings). Given the circumstance that MP had been involved in the development of the SELFIE intervention, MP did not actively participate in the focus group as a participant, nor as a leading host of the focus group. SV led the focus group and MP was present mainly in taking notes. All the stakeholders were questioned about the purpose of the SELFIE intervention, key aspects of the program, its implementation, how they believe the program should work, and the expected outcomes. The individual expert interviews and the focus group were audio recorded and transcribed verbatim.
*Analyses*	Phase 1 of the current realist evaluation entailed the development of an IPT. Identifying key elements of contexts and generative causation guided the analysis. ‘Context’ was interpreted as a circumstance (e.g., personal characteristics, cognitive capacities, or interpersonal relationships) that can activate, promote, or inhibit the activation of a mechanism. ‘Mechanism’ was defined as an explanatory theory of what causes changes in individual behavior [38]. The analysis consisted firstly of open coding, aiding in limiting possible premature interpretation by the researchers. Consequently, basic codes were merged into more abstract groups or categories (axial coding). During the process of analysis, through regular discussion consensus was met by researchers SV and MP, and consultation on realist methodology was offered by LD and MS who were not directly involved in the SELFIE trial. MP finalized the analysis of phase 1. Preliminary clustering of the data under context, mechanism, and outcome, and consequently interpretation in the context of literature, led to the construction of overarching program theories. In phase 1 the program theories were stated as “If…., then… - statements”, adhering to recommendations to start with broad program theories within complex intervention evaluations and to construct context-mechanism-outcome-configurations (CMOCs) at a later stage [64]. This supported adhering to a broad view when analyzing the data and thus facilitated openness to non-confirming insights. The language used in all the transcripts of this realist evaluation, apart from the stakeholder’s focus group, is Dutch. This language was also used in the process of analysis, and only for the cause of presenting quotes in this manuscript, translations were made. Atlas.Ti software was used to support the process of analysis [65].
***Phase 2 (testing the IPTs)***	
*Participants*	Participants in the study who had received the SELFIE intervention were included in the current realist evaluation after completing the SELFIE intervention but before the six-month follow-up. Inclusion criteria were equal to those applied in the SELFIE trial: youth between 12 and 26 years of age; having experienced adverse childhood events (one or more of the following: physical abuse, sexual abuse, emotional abuse, emotional neglect, physical neglect, bullied or have witnessed domestic violence between parents), assessed with the Childhood Trauma Questionnaire (CTQ) [66], Retrospective Bullying Questionnaire (RBQ) [67], and the subscale Parental Conflict of the Childhood Experience of Care and Abuse Questionnaire (CECA) [68]; reporting low self-esteem (a score below 26 on the Rosenberg Self-Esteem Scale (RSES) [69].
*Recruitment and data collection*	Participants in the SELFIE trial gave consent before randomization and could indicate whether or not they approved of being contacted for future research. Even though the realist evaluation is part of the SELFIE trial, we adhered to contacting only those participants who had approved at initial inclusion to limit undesired burden. We followed the maximum variation sampling method, followed by a pragmatic sampling strategy, to include participants from different (clinical) settings, to the extent this was possible within the time frame of the realist evaluation as well as the relatively small sample size. Participants were informed about the process evaluation (referred to this realist evaluation as process evaluation because of the familiarity of this term to participants) and asked if they would like to participate. If so, they received an information letter on the process evaluation and after a one-week reconsideration period, they were contacted again. Once a participant agreed to participate and written informed consent was given, a meeting for the interview was scheduled. Fifteen interviews took place. Due to COVID-19 restrictions, all interviews were held online through video meetings, and the interviews lasted on average 60 min. Findings from phase 1 in combination with literature, informed the development of a topic guide for the semi-structured interview with participants, and the first interviews informed minor adjustments to the topic guide (see Supplementary material 4). After completion of the interview, the participant received compensation of €10 in their bank account. In addition to the interviews, data was extracted from a debriefing questionnaire. All participants in the experimental group of the SELFIE trial were asked to fill in a debriefing questionnaire directly following their participation in the SELFIE intervention, inquiring about their experiences with the SELFIE intervention. Of all participants (*n* = 85) in the experimental group of the SELFIE trial, 61 participants completed the debriefing questionnaire. Data was saved anonymously on a secured server. This debriefing questionnaire data was used complementary to the interviews to test the IPTs. Furthermore, SELFIE therapists were contacted for participation in a focus group. It was hypothesized that therapists, based on their experience with multiple participants, could inform the process evaluation on aspects of context. Six SELFIE therapists (excluding researchers) delivered the intervention more than three times. After contacting them they received information on the process evaluation, were given a week to decide, and after written informed consent was given the focus group was scheduled as a video meeting, which 4 SELFIE therapists attended. MP led the focus group based on a topic guide that was informed by findings from the interviews with participants (see Supplementary material 5), and KS observed the interaction and made notes. The SELFIE therapists were not compensated for their participation in the focus group. The interviews aimed to test the IPTs [70], and were audio-recorded and transcribed by the researchers. Data was saved anonymously on a secured server.
*Analyses*	Data collection and analyses were part of an iterative process. Data from three interviews were analyzed by open coding at first by (partly joint) two researchers (KS and MP) to set up a code-book, this code-book led the future coding whereby data describing generative causation was extracted. Atlas.Ti software [65] was used in this process. The coding was done individually, however, a total of five interviews were coded by both KS and MP and discussed to enhance intercoder agreement, consistency, and neutrality of evidence [63]. Research partners KS and MP met regularly during the process of data collection and analysis, for discussion and comparison of collected data, to review all quotations, to reach further consensus about coding, insights, and inferences, and in concordance, CMOCs were formulated. Analysis of the debriefing questionnaire was done in parallel.

### Participants

Phase 1 (the development of the IPTs) was based on literature study, qualitative data from 2 expert interviews, and a focus group with stakeholders. Subsequently, for phase 2 (testing the IPTs) iterative data collection took place through 15 individual interviews with SELFIE participants (within six months after finishing the intervention), analyzing data from a debriefing questionnaire (*n* = 61), and a focus group with 4 SELFIE therapists. As stated, more details of the methods used in recruitment, data collection, and analyses are shown in Table 1.

### Analyses

As stated above and shown in Table 1, data collection and analyses took place in an iterative process during the different phases of this realist evaluation leading to the development of IPTs after phase 1 and formulated context-mechanism-outcome configurations (CMOCs) after phase 2. In parallel, a debriefing questionnaire (participants filled this in directly after receiving the SELFIE intervention, and it inquired about participants’ experience with the app, the use of the exercises, satisfaction, and acceptance of the SELFIE intervention) was addressed in testing the IPTs. In phase 3 these CMOCs were synthesized back into the IPTs. MP conducted this analysis individually, and subsequently discussed it within the research team. The process of analysis was inspired by the approach described by Gilmore et al. [51]. Namely, IPTs were coded as nodes, to which quotations were assigned illustrative for IPT-specific CMOCs and their implications for the IPTs (refute, refine, or accept). To ensure transparency, the process of reasoning was explicitly described in research memos.

## Results

### Stakeholders and participants

For phase 1, two experts were contacted and found willing to participate. Further, four stakeholders took part in a focus group. For phase 2, firstly 23 participants in the SELFIE trial were contacted of whom 15 took part in semi-structured online (due to COVID-19 restrictions) interviews.

**Table 2:** Participant characteristics of the interview sample of this RE and the participant characteristics of the full sample of participants in the experimental condition of the SELFIE trial

Characteristic	Full sample experimental condition RCT	Total no.	Interview sample	Total no.
Age, mean (SD), y	20.86 (3.00)	85	21.54 (2.50)	15
Sex, No. (%)		85		15
Female	73 (85.88)		12(80)	
Male	11 (12.94)		3 (20)	
Other	1 (1.18)		0 (0)	
Study center/route into study, No. (%)		85		15
Noord-Holland	6 (7.1)		0 (0)	
Zuid-Holland	11 (12.9)		2 (13.33)	
Limburg	26 (30.6)		5 (33.33)	
General population	42 (49.4)		8 (53.33)	

*Note* S.D., standard deviation

Table 2 shows the characteristics of this interview sample alongside the characteristics of the full sample of participants in the experimental condition of the SELFIE trial [31]. Not having sufficient time (*n* = 6) and mentally not feeling well enough to participate (*n* = 2) were reported as reasons not to participate. The average age of the participants was 21 years; 12 participants identified as women, and three as men. Secondly, six therapists who delivered the SELFIE intervention were contacted of whom four participated in a focus group. Non-participation was due to agenda restrictions (*n* = 2).

### Main findings phase 1 (developing the IPTs)

Three pillars of the SELFIE intervention were defined based on its program architecture. First, it is delivered as an EMI, second, the intervention aims to target self-esteem, and last, it is offered as a guided self-help intervention. As described before, the working mechanisms that underpin EMIs are not fully understood, and this study seeks to obtain data that will help address this research gap by focusing the present analysis solely on the pillar regarding EMI. The other pillars are discussed in a separate paper [50].

Data collected during Phase 1 was described and interpreted in the context of pre-existing literature and theories. A detailed description of the process of identifying the IPTs can be found in Supplementary Material 6.

The three key IPTs related to EMI that were identified, tested against our data, and refined, were:

- If participants experience the intervention as personalized, anonymous, and easily accessible, then they will be more comfortable with their input and can participate in the SELFIE intervention without feelings of shame.’ (IPT 1).
- If a change in momentary self-esteem is established repeatedly and under different circumstances through the use of a smartphone application, then this will support the generalizability of this effect and support change in general self-esteem.’ (IPT 2).
- If a technical malfunction is present or the reminder beeps are being perceived negatively, then the effect of the intervention will be limited and drop-out may occur.’ (IPT 3).

Table 3 shows the initial program theories, with supportive literature.

**Table 3:** Initial Program Theories on how the SELFIE intervention as an EMI may exert its effect on most users

Initial Program Theory	Supporting theories from the literature
Ecological momentary intervention	
1. If participants experience the intervention as personalized, anonymous, and easily accessible, then they will be more comfortable with their input and can participate in the SELFIE intervention without feelings of shame.	EMIs are available when it is most needed [11, 71]. Furthermore, advantages of this intervention method have been reported such as increased accessibility of treatment and the possibility to give personalized feedback and support [60]. Findings suggest that anonymity and privacy offered by a digital intervention, are highly appreciated by users [10]. Besides privacy, digital interventions may further provide comfort, and acceptance of the intervention [72]. Using a smartphone application is suggested to be convenient and easily accessible, but also private and offers the possibility to engage without experiencing (self)stigma, possibly enhancing motivation to participate [73]. The importance of tailoring interventions based on individual needs has been emphasized [24], and non-tailored EMIs are less well perceived by users [14].
2. If a change in momentary self-esteem is established repeatedly and under different circumstances through the use of a smartphone application, then this will support the generalizability of this effect and support change in general self-esteem.	Evidence for the effectiveness of cognitive-behavioral therapy is substantial, however, the effectiveness seems to be limited regarding the generalization of treatment effects to the real world of patients [67, 68, 69]. Incorporating mobile technology into mental health interventions offers a means to reinforce the systematic use of treatment components in real-world settings, thereby enhancing the generalization of the impact of the intervention [14, 74]. Ecological Momentary Interventions (EMI), because being delivered in individuals’ natural environments, offer the opportunity to apply new skills and behaviors in their real-world experience. Research indeed suggests (personalized) EMIs support the generalization in daily life [75]. Mobile technology aids the effective delivery of an EMI because its usage and interest are widespread, especially among youngsters. This increases feasibility for usage during daily life [76].
3. If a technical malfunction is present or the reminder beeps are being perceived negatively, then the effect of the intervention will be limited and drop-out may occur.	A systematic review of digital mental health interventions for depression and anxiety in young people presented low rates of engagement and adherence, whereby technical malfunction was described as an influential factor [10]. The concern of technical problems was also addressed by Donker et al. [60]. Findings related to the potentially disruptive experience of reminder beeps showed participants usually appreciated the tool but may need time to adjust to these beeps [77].

### Main findings phase 2 and 3 (testing and refining IPTs)

The IPTs as shown in Table 3 were tested (phase 2) and refined (phase 3). Our main findings will be described below per IPT in the form of CMOCs as the analytical template of realist evaluation, followed by the refined IPT.

#### Testing IPT 1

A reported response to the intervention being offered repeatedly in daily life and being constantly available was to experience the intervention as accessible and feasible due to a lack of travel time to appointments, short exercises, reminders on the phone, and the ability to practice an exercise when there is time and need. The following quote illustrates the availability of the smartphone application being offered on their personal phone (important context vs. receiving a study phone).

*“Ehm, well yes, it is nice that you can fill it in wherever, it does not matter where you are. I mean, yes ok, you could also bring a booklet anywhere but that would be something you might forget now and again. But you will not forget your phone as easily. You would go back home for it if you would forget it. So ehm, if you are on the train, at work, or you have a short break, then you can just work on it [the SELFIE intervention].* – Source: PI_3 (356–360)

Offering the intervention on their personal smartphone enhanced active participation, as illustrated in the following quote, supporting intervention outcomes.

“*Yes, well it is just easy because you always carry your phone with you. So you don’t have to make a great effort (…) if I would have to do it [the exercises] on the computer, which I don’t carry with through the entire day, then you have to especially sit down to do it, making time for it, I feel. And with the app, you can note things down in between moments when you have some time left. (…) Yes, I think [that if I would have to use a computer] I would have been less engaged with the intervention. Because of the app, it just offers you easy access and so on, and that made me participate more actively than if I would have done it on a computer*.” – Source: PI_6 (196–205)

In testing IPT 1, data supports the notion of ‘easily accessible’ as stated in IPT 1. Furthermore, it offers more detailed insight into why it is experienced as accessible. Having an intervention on your smartphone helps youngsters to stay committed due to the constant availability of the intervention, aiding flexible use of the exercises and making it more feasible to integrate it into their daily life since the expense in time is limited due to a lack of travel time to appointments for example. Supportive data was extracted from a debriefing questionnaire in that participants report to have experienced that the application helped them to practice the exercises in daily life, and that during the day they repetitively were aware of their context, feelings, thoughts, and behavior (as can be seen in Table 4).

**Table 4:** User experience and acceptance of the SELFIE intervention (*n* = 61)

User experience, mean (S.D.)*	
Did the PsyMate help you to apply theexercises in your daily life?	5.13 (1.18)
To what extent have you been aware of your situation, feelings, thoughts, and behavior, for several times a day?	5.48 (1.07)
To what extent did the following elements contribute to a change in your self-esteem:	
Exercises via app	4.65 (1.57)
Availability of exercises at all times	4.62 (1.44)
Sessions with therapist	5.33 (1.39)
Email contacts	3.95 (1.60)
Did you come across certain problems when using the PsyMate?	3.45 (1.66)
Have there been moments when you experienced the use of the Psymate as annoying?	3.15 (1.55)
Satisfaction with SELFIE intervention, mean (S.D.)*	
Was the SELFIE intervention useful for you?	5.23 (1.35)
Were the homework exercises useful?	5.22 (1.34)
Were the face-to-face-sessions useful?	5.42 (1.37)
Was the guidance within the SELFIE intervention sufficient?	6.12 (0.96)
Was the SELFIE intervention applicable to your symptoms?	5.13 (1.64)
Acceptance of the SELFIE intervention, mean (S.D.)*	
To what extent are you convinced of the effect of the SELFIE intervention?	5.03 (1, 43)
Did you experience this way of receiving help (sessions with a SELFIE therapist and individual exercises in the app) as pleasant?	5.53 (1.43)

*Note* S.D., standard deviation

* Rating on a scale of 1 to 7

Regarding the sense of anonymity as phrased in IPT 1, it was found that the exercises were offered individually and privately, which offers a sense of privacy and anonymity without the interference of (possible) responses of others (response). The following quote from a participant illustrates this experience.

*“By writing it down yourself in an app dares me to write down more that when I would have to say it (…) Yes, the idea that you are more anonymous, that people will not directly see or read everything or, well yes if you are in a conversation with someone else, I would experience that stronger. Now it was more the idea of writing it for yourself, rather than for someone else” -* Source: PI_6 (182–195)

The sense of privacy and anonymity led to participants being less hesitant to fill in exercises in an honest and ‘unfiltered’ manner.

Reflecting on IPT 1, elements of ‘anonymity’ and ‘feeling more comfortable with their input’ are supported by our data. However, regarding the experience of participants that others will not interfere with their input, the word ‘privacy’ seems to be a better fit for this experience than ‘anonymity’ (as was used in IPT1). Participants were aware that their SELFIE therapist was able to partly read along on a reporting page, thus it wasn’t completely anonymous in that sense. In summary, a sense of privacy adds to the openness of participants when engaging in the intervention. They are less hesitant to fill in answers due to a lack of response by another person (for example, questioning their input further, or having an opinion about their input). It was inferred that open and active participation in the intervention is supportive for the intervention outcomes relating to self-esteem.

#### Refined IPT 1

The aforementioned findings in testing IPT 1 have been framed as CMOCs, as depicted in Table 5. These CMOCs have informed the following refinement of IPT 1:

- The SELFIE intervention is offered through a smartphone application (C) enabling constant availability of the intervention and thereby increasing accessibility and feasibility (M), and, when offered on their personal smartphone (C) this enhanced a sense of privacy ensuring less hesitance in engaging with the app (M), leading to more open and active participation (O).

**Table 5:** CMOCs relating to IPT 1

Context	Mechanism	Outcome
Smartphone application	Intervention is offered repeatedly in daily life and is constantly available (resource) – due to a lack of travel time to appointments, short exercises, reminders on the phone, and the ability to practice an exercise when there is time or need, the intervention is experienced as accessible and feasible (response)	More active participation and thereby supporting intervention outcomes
Intervention is offered through a smartphone application	Exercises are offered individually and privately (resource) – experiencing a sense of privacy and anonymity without the interference of (possible) responses of others (response)	Less hesitant to fill in exercises in an honest and ‘unfiltered’ manner.

#### Testing IPT 2

Generalizability of effect is reported, however, emphasis is placed on acquiring a non-situation-specific skill (not dependent on situation-specific characteristics), and thereby able to lead to the generalizability of effect. The following quote refers to an exercise aimed at coping with receiving criticism, in which the participant previously mentioned that due to the exercise she gained a new perspective:

*Interviewer: (…) What happens consequently when you look at things differently, does that change something in your thoughts or your feelings? What is its effect?*

*Participant: Mainly in my thoughts, that I think ‘ok’, well if I take the example again of that exercise on criticism, then, yes, how do you say that?… All of a sudden you are not so afraid of receiving feedback anymore or criticism or something like that, because I…, it has never been proven but I do think that I have a fear of failing. So I always found it very scary to make mistakes. And if it happens now I think ‘Yes, but actually, there is nothing wrong’. Because of that I then feel a lot more confident in my actions and experiences. Not just at work, but also in daily life. Well… my feeling related to that, that could make me very sad, that I felt that way, I could also be very anxious when I received criticism, which really frightened me. But, that is now a lot less.”* - Source: PI_15

Participants rated the offering of prompting beeps for and the constant availability of the exercises as more than moderately effective in aiding change in self-esteem (also shown in Table 4). The sessions with the SELFIE therapist were rated slightly higher in supporting change in self-esteem, possibly implying the importance of guidance when delivering an EMI.

Synthesizing the above findings back into the IPT, we inferred that for the SELFIE intervention repetition in daily life is key in creating the circumstance of being more prone to cognitive restructuring, and since this surpasses situation-specific characteristics, with additionally the availability of certain tailored situation-specific exercises (e.g. criticism exercise), it could be argued that the effect is generalized over different situations in daily life. Thus generalizability is enhanced through the offering of the intervention in daily life as well as the availability of tailored interventions (as stated in IPT 2), however, this is not through the mechanism of changing momentary self-esteem in different situations, rather the mechanism seems to be that cognitive restructuring is more prominent and not dependent on situation-specific characteristics whereby effects are generalized.

#### Refined IPT 2

The above findings are phrased as a CMOC (presented in Table 6), and led to the following refined IPT 2:

- Offering the intervention through the use of a smartphone application (C), facilitates the practice of skills that are not dependent on situation-specific characteristics in daily life, supporting repeated practice in different situations (M), leading to the generalizability of the effect (O).

**Table 6:** CMOC relating to IPT 2

Context	Mechanism	Outcome
Well trained skills that facilitate cognitive restructuring	Aid in making use of offered tools in daily life to view circumstances as more positive and support a less negative mindset in different situations thus facilitating a change that is not restricted to certain situations,	And leads to confidence and mental well-being generalized to different situations in daily life.

#### Testing IPT 3

Reminder beeps for the intervention exercises were offered through the smartphone application, popping up at random moments with an alerting sound. In some cases, as for the participant sharing the below-placed quote, this was experienced as loud and disruptive and/or for the participant at inconvenient timing.

*“Ehm, yes I think the beeps are not so pleasant, I sometimes experienced them as rather annoying actually. (…) it beeped at inconvenient times, and the sound was also quite loud. Yes, it startled me sometimes .”* - Source: PI_4 (317–321)

Thus, reminder beeps may sometimes be perceived as annoying or intrusive by sound or timing. Our data showed this could lead to negative sentiment and actions, such as turning off notifications and sound, with subsequently less active participation. It seems to be a balancing act between the intensity (repetition and duration) of the intervention being sufficient to reach positive outcomes (our data showed that in most cases, the reminder beeps were perceived as a positive element in supporting active participation), and the intensity being experienced as too much of a burden. Personal circumstances (well-being, energy, focus) seem to be an important context regarding assessing burden by capacity.

In the context of the intervention being delivered in the form of a smartphone application, technical malfunction may arise to which participants may respond by experiencing ‘hassle’ accompanied by irritation and a decrease in motivation. The following quote from a SELFIE participant who received the intervention illustrates discontinuity.

*“It just made me postpone when it didn’t function to log in with the code, and yes, when I would receive a new code I would be in school for example, and with getting the email [with the new code] I would think ‘yes, I will do that this evening’, or something. (…) that made that actually one day or so was ‘lost” –* Source: PI_1 (514–518)

In testing IPT 3, our data support the notion of a technical malfunction being perceived negatively and leading to less effective participation or drop-out. When, as part of the debriefing questionnaire, participants where asked about any problems they faced when using the SELFIE intervention, or whether they experienced it as annoying, rated as rarely (find participants’ ratings in Table 4), they mainly reported technical issues to explain their rating. In some cases, it was explained by their experience of not having enough space in the text boxes to write their answers. Technical malfunction is mainly experienced as a burden since it encompasses extra moments of contact or actions, both participants and SELFIE therapists have reported this. If needed, technical assistance was mentioned by participants to be readily available through, among more, WhatsApp, which was experienced as a low threshold, which is an important context for IPT 3. This buffered against the negative effects of technical malfunction. Further, as is shown in Table 4, ratings on the debriefing questionnaire prove very good satisfaction with and acceptance of the SELFIE intervention when also taking into account the mode of delivery.

#### Refined IPT 3

The aforementioned reflections have led to developing CMOCs relating to IPT 3, which can be found in Table 7. Subsequently, these CMOCs have informed the following refinement of IPT 3:

- The use of a smartphone application to deliver the SELFIE intervention (C) may encompass technical malfunction and accompanied irritation and demotivation (M), leading to less active or delayed participation (O).
- Furthermore, the reminder beeps (C) are activated at random moments and with an alerting sound which can be experienced as loud and disruptive (M), leading to decreased motivation and less active participation by e.g., turning off the notification or sound (O).

**Table 7:** CMOCs relating to IPT 3

Context	Mechanism	Outcome
The intervention is delivered through a smartphone application	Technical malfunction (resource) – frustration due to experiencing this as ‘hassle’ and a decrease in motivation (response)	Less active participation or delayed participation due to lack of immediate resolvement of problem (by either SELFIE team or participant)
Reminder beeps offered through a smarthone application	Popping up at random moments with an alerting sound (resource) – experienced as loud and disruptive and/or for the participant inconvenient timing (response)	Turning off notification or sound and experiencing decreased motivation and subsequently less active participation

## Discussion

### Summary of findings

The aim of the present study was to investigate, within the SELFIE intervention, working mechanisms of EMI, and under what circumstances these working mechanisms do or do not come into play. The current research led to a revision of three IPTs. First, “The SELFIE intervention is a blended therapy using a smartphone application (C) enabling constant availability of the intervention and thereby increasing accessibility and feasibility (M), and, when offered on their personal smartphone (C) this enhanced a sense of privacy ensuring less hesitance in engaging with the app (M), leading to more open and active participation, thereby supporting intervention outcomes (O).” This refined programme theory illustrates that a smartphone application supports the mechanism of constant availability and consequently increases accessibility and feasibility. A supporting context for this mechanism is the use of their personal smartphone (rather than receiving a study phone), enhancing active participation and responding to the easily accessible intervention. Second, “Offering the intervention through the use of a smartphone application (C), facilitates the practice of skills (i.e. cognitive restructuring) that are not dependent on situation-specific characteristics in daily life, supporting repeated practice in different situations (M), leading to the generalizability of the effect (O).”. This refined programme theory stresses the importance of intervention exercises matching a variety of situations, meaning that if an intervention is offered repeatedly and in different situations, it will not be effective if the exercise offered is not applicable in a given situation. For the SELFIE study, it was found that practicing a relevant skill was not dependent on situation-specific characteristics and thereby effective to be offered repeatedly over a variety of situations. In addition, situation-specific exercises (such as how to deal with criticism) could be offered when permitting availability on demand. Third, “The use of a smartphone application to deliver the SELFIE intervention (C) may encompass technical malfunction and accompanied irritation which may induce demotivation (M), leading to less active or delayed participation (O).” Furthermore, “the reminder beeps (C) are activated at random moments and with a loud alerting sound which can be experienced as disruptive (M), leading to decreased motivation and less active participation by e.g., turning off the notification or sound (O).” These refined programme theories show that technical malfunction and disruptiveness of beeps do decrease motivation and engagement with the intervention. Since these ‘hurdles’ will not completely cease to exist, it is important to ‘buffer’ against the negative effects of this when delivering an EMI. We found that technical assistance being readily available and matching the participants’ needs (in this case youngsters who responded well to WhatsApp), may buffer against the negative effects of technical malfunction.

### Comparison with existing literature

Delivering the SELFIE intervention through a personal smartphone app was found to be supportive of the outcome (refined IPT 1), which is in line with previous findings that the use of a smartphone seems to be associated with higher rates of adherence [52]. The use of one’s smartphone and not requiring multiple devices is often described as supporting the accessibility and usability of an EMI [53]. The current study sheds more light on the experience of youth in using their smartphone, not only supporting usability through convenience as previously reported but also revealing an underlying sense of privacy which seems to make participants less hesitant to engage with the app and thereby supporting open and active participation. Furthermore, the mentioning of a sense of privacy by participants links to research on therapeutic relationships in digital mental health delivery. A process proposed to enhance the openness of patients has been described as disinhibition, whereby clients were more prone to disclosure than in regular face-to-face contacts [54]. This is consistent with research on assessment methods, with computer-administered assessment methods obtaining more honest, open responses of personal information [55]. Furthermore, contact with a ‘virtual human’ controlled by a computer seemed to support participants’ openness and self-disclosure due to the experience that their responses were not being judged by another human, with the underlying assumption that experiencing fear of judgment will activate impression management resulting in withholding information that might threaten their reputation [55]. In contrast, even though quantitative research on the effect of coaching within an app-based intervention is limited and somewhat diffuse [56], evidence does seem to point towards human guidance within an app-based intervention to be positively associated with engagement, completion rates, and treatment outcomes [10, 57–59], and offering smartphone apps as standalone psychological interventions is not advisable due to the low level of current evidence on this [35]. These findings corroborate the results of the previous realist evaluation within the SELFIE trial, researching the element of guided self-help [50]. Thus, clinical judgment regarding the intention of an EMI could inform decision-making in EMI development on the degree of human interaction. The afore-described illustrates that an EMI with solely digital components is likely to differ substantially from an EMI with additional guidance by a therapist, and thus the findings from the current research should be placed within the context of guided EMIs.

The current results in the form of refined IPTs tentatively support the ecological interventionist causal model [12] in that participants report having practiced the intervention in their daily lives and that in their perception it leads to changes regarding the target mechanism of self-esteem. This is supportive of the main findings from the SELFIE study showing improvement in the primary outcome of self-esteem at post-intervention and 6-month follow-up [23]. As mentioned in the introduction, EMIs can prompt contextual reminders to trigger specific behaviours in specific contexts [24, 40, 41] aiding change in the aspired outcome of an intervention. In addition to this existing literature, our findings suggest exercises not only to be customized at the moment (and thus being context-specific) but also offer exercises to facilitate the practice of skills that are not dependent on situation-specific characteristics in daily life. By supporting repeated practice in different situations an EMI may broaden its relevance for the user.

In accordance with the current refined IPTs, previous studies have demonstrated that technical malfunction may be a negative influential factor regarding adherence and satisfaction when using app-based interventions [10, 60]. Furthermore, the finding that electronic prompts could be experienced as disturbing broadly supports the work of other studies where electronic prompts were experienced as a burden when they requested assessments of length, and a higher number of missing answers were reported when participants were prompted 8 times a day or more [52]. Thus, the findings replicate existing knowledge on prompts and technical issues with EMIs, however, the notion of a ‘buffer’ against possible negative effects by specifically adhering to the preferences of participants (in the current study the participants reported WhatsApp to be a low-threshold way of communicating, in contrast to email) is novel and further research may serve effective implementation and adherence to an EMI. User-experience outcomes could aid this type of research [61].

### Strengths, limitations, and future directions

The method of realist evaluation made apparent possible working mechanisms of EMI which, even though researched within the context of the SELFIE intervention, seem to be relevant for other forms of (guided self-help) EMI. This is of crucial importance in the current state of research in the field of EMI, since developing EMIs in itself should be driven more by existing knowledge and theory of the working mechanisms to provide enhancement of the field. We would suggest that every trial that tests the efficacy of an EMI adds a process evaluation to interpret the main findings of the efficacy trial as well as to build knowledge on the working mechanisms of EMIs for further development and implementation. The current research offers insight into underlying processes that may exert an effect on the main outcome findings of the SELFIE trial. It should be noted, however, that the interactive element within the SELFIE intervention has not been addressed sufficiently concerning its importance within EMIs in the current paper. We would therefore suggest future research on this particular aspect. Further, synthesizing findings on efficacy in the SELFIE trial with findings from the realist evaluation would be of added value to deepen our understanding of interventions as well as target mechanisms as in this case self-esteem, and therefore, such a mixed-methods design would be strongly suggested for future research.

A future recommendation for EMI may be to allow participants to choose the notification sound and allow more flexibility in choosing time blocks. Studies using a pre-fixed sampling scheme (the prompts were programmed at certain times per day, and if multiple prompts were programmed, a fixed time interval was used) reported higher rates of adherence in (EMA) studies [52], however these studies offered very little prompts per day and did not aim to capture variability over the course of a day. The use of a pre-fixed sampling scheme is of course a trade-off with the effects due to the random occurrence of beeps and would not fit the aims of the SELFIE intervention since it would impair representative characterization of experience and, hence, limit EMIs substantially in tailoring/adapting to moments when help is most needed. Therefore, for the SELFIE intervention, we would recommend to offer flexibility in choosing the time blocks of a random sampling schedule within set blocks of time.

Furthermore, findings from feedback from users in an EMI for major depressive disorder reported three EMI features to have been highly appreciated: the possibility of receiving visual feedback about daily assessments, and consequently self-monitoring of daily patterns; the availability of psychoeducational material on depression and its mechanisms; and the opportunity to have continuous or periodic communication with a trained clinician [30]. Considering that the SELFIE intervention provides communication with a trained clinician, as well as psychoeducational material on self-esteem as session content, a new implication of the afore mentioned findings of Colombo, Fernández-Álvarez [30] is that the SELFIE intervention may benefit from offering participants visual feedback. In further development and implementation of the SELFIE intervention, the actual end users should continue to be involved in the design and evaluation of the technology for it to match their needs and to ensure that engagement is maximized [62].

Regarding methodological concerns, along with her role as researcher, MP was involved in developing and delivering the SELFIE intervention, which may lead to bias in carrying out the realist evaluation. To ensure reflexivity toward her role as a researcher, regular meetings with the research team were held, thereby focusing on the quality criteria of neutrality. Furthermore, the consensus in decision-making during the research process as well as the analysis was fostered through assistance by two other researchers (SV and KS) employing investigator triangulation over the study period. In addition, researchers not directly involved in the SELFIE trial (LD and MS) were regularly consulted. In contrast, the role of MP as being involved in both delivering and researching the SELFIE intervention may have supported the quality principle of applicability of evidence through transferability, i.e. the extent to which the current findings can be transferred or applied in different clinically relevant settings [63]. Second, despite applying a maximum variation sampling method, as described in Table 1 under ‘phase 2’, selection bias may have occurred in that individuals with particular characteristics might have agreed to participate in the study (e.g., participants experiencing enthusiasm regarding the SELFIE intervention may be more willing to share their experiences). For the focus group with SELFIE therapists, 4 out of 6 invited therapists were able to join the focus group. The two who were not able to attend reported this was due to agenda constraints and no particular characteristics of these two are thought to have had a bearing on the focus group data. Third, the timing of interviewing the participants was within six months after finishing the intervention to ensure a sufficient amount of interviews. It should be noted that the ability to recall the exact exercises and personal experiences is limited over such a period and should be taken into account when interpreting the findings. Future research should aim to interview participants closer to the date of finishing the intervention. Lastly, even though it is expected that certain findings are generalizable for EMIs not targeting self-esteem, it should be taken into account that self-esteem in itself may be a very important context for certain EMI working mechanisms to exert its effect. Illustrative of this is that our data support the notion that EMIs are particularly supportive of skill development. Furthermore, the context of the present study was a guided self-help intervention, this may limit the generalizability of our findings to EMIs in general. Therefore, the earlier-mentioned suggestion to promote research on the working mechanisms of EMI could aid in identifying certain combinations of context and working mechanisms to be effective.

## Conclusions

The current research addressed working mechanisms of an EMI targeting self-esteem, such as the constant availability supporting increased accessibility and feasibility, for which the use of the personal smartphone was experienced as a facilitating context. Further, findings stressed the importance of delivering non situation-specific exercises (to support cognitive restructuring), or having situation-specific exercises available on demand (to aid in practicing adaptive copingstrategies in a given situation), to facilitate the mechanism of generalizability and ecological validity as an appreciated aspect of EMI. Lastly, technical malfunction and the burden of electronic prompts were experienced, reflecting previous literature, indicating the need for further research on ‘buffering’ possible negative effects of technical malfunction when delivering EMIs. Interestingly, within the SELFIE intervention, the context of accessible technical assistance showed indeed to be such a buffer against the negative effects.

The present findings can help us to understand possible working mechanisms and their contexts of EMIs, contributing to relatively limited research in this field. For the field to move forward, a better understanding of the working mechanisms of EMI needs to be developed, and it is strongly recommended that alongside efficacy trials of an EMI on specific target mechanisms, a process evaluation is conducted investigating the working mechanisms.

### Electronic supplementary material

Below is the link to the electronic supplementary material.

Supplementary Material 1

## Data Availability

The data that support the findings of this study are available from the corresponding author upon reasonable request.

## Abbreviations

CMOC: Context-mechanism-outcome configuration
EMA: Ecological Momentary Assessment
EMI: Ecological Momentary Intervention
ESM: Ecological Sampling Method
IPT: Initial programme theory
JITAI: Just-in-time adaptive intervention
