# Supplementary material for: Working mechanisms of the use and acceptability of ecological momentary interventions: a realist evaluation of a guided self-help ecological momentary intervention targeting self-esteem
Source: BMC Public Health. 2024 Jun 19;24:1633. doi: 10.1186/s12889-024-19143-z (PMC11186172; doi:10.1186/s12889-024-19143-z)
Supplement: Supplementary file 1 — Supplementary Material 1 [file 12889_2024_19143_MOESM1_ESM.pdf]

**Supplementary material 1**

*Topic-guide interview expert on self-esteem interventions*

SELF-HELP APPROACH (The SELFIE intervention was partly based on the book: 'Build your confidence with CBT')

- What changes are you trying to accomplish with your self-help book intervention'?
- What do you think is the working mechanism of the self-help intervention? And has this changed over time, for example, based on the comments of participants?
- What could work against obtaining desired outcomes?
- What cognitive behavioral interventions are being offered?
- Have other elements been added to the book (apart from cognitive behavioral elements) which could have exerted an effect on self-esteem?
- Would you have an idea of which circumstances affect the desired outcome? Examples?
- Do you think that specific elements of the self-help intervention are tailored for youth, and which elements are not?

SELFIE

- Within the SELFIE intervention, exercises from your self-help intervention are being offered through a smartphone application. How do you expect that to have an effect? What changes (or doesn't) according to you?
- What in your opinion is the effect of 'shaping' and 'modeling' if practiced by SELFIE therapists who offer the SELFIE intervention?
- Regarding facilitators and barriers, which do you believe to be relevant when offering an intervention targeting self-esteem through a smartphone application?
- What is needed to make a smartphone application targeting self-esteem successful for youth?
- Discuss the difference in the time frame of intervention: the self-help book suggests taking 6 months to go through it, and the SELFIE intervention encompasses 6 weeks. What are your thoughts on this?

IN GENERAL

- Do you feel there is a gap between youth experiencing low self-esteem, and interventions aimed at targeting self-esteem? If you do, what is important to bridge that gap? If not, what makes this well-adjusted?

**Supplementary material 2**

*Topic-guide interview expert on ESM/EMI*

EMI

- What changes are you trying to accomplish with an EMI? And in what manner (what do you see as the working mechanism of an EMI)?
- Would you have an idea of which circumstances affect the desired outcome? Examples? (context)
- What could work against obtaining desired outcomes?
- Do you think that specific elements of an EMI are tailored for youth, and which elements are not? (context)

SELFIE

- What do you think of the combination of an EMI and self-esteem, in what way is that a good match or not?
- SELFIE is being offered as guided self-help. We ask therapists to be aware of cognitive behavioral techniques, such as 'shaping' and 'modeling'. What do you think is the effect of encouraging SELFIE therapists to make use of 'shaping' and 'modeling'? Is that of added value, or not, in combination with an EMI?
- Regarding facilitators and barriers, which do you believe to be relevant when offering an intervention targeting self-esteem through a smartphone application?
- What is needed to make a smartphone application targeting self-esteem successful for youth?

IN GENERAL

- Do you feel there is a gap between youth experiencing low self-esteem, and interventions aimed at targeting self-esteem? If you do, what is important to bridge that gap? If not, what makes this well-adjusted?

**Supplementary material 3**

*Topic guide for the focus group with stakeholders*

SELFIE

- What change is the SELFIE intervention trying to accomplish?
- And in what manner (what do you see as the working mechanism of the SELFIE intervention)?
- What do you think are important circumstances to affect the desired outcome of the SELFIE intervention? Examples?
- What could work against obtaining desired outcomes?
- Do you think that specific elements of the SELFIE intervention are tailored for youth, and which elements are not? (context)
- SELFIE is being offered as guided self-help. We ask therapists to be aware of cognitive behavioral techniques, such as 'shaping' and 'modeling'. What do you think is the effect of encouraging SELFIE therapists to make use of 'shaping' and 'modeling'? Is that of added value, or not, in combination with an EMI?
- Regarding facilitators and barriers, which do you believe to be relevant when offering an intervention targeting self-esteem through a smartphone application?
- What is needed to make a smartphone application targeting self-esteem successful for youth?

IN GENERAL

- Do you feel there is a gap between youth experiencing low self-esteem, and interventions aimed at targeting self-esteem? If you do, what is important to bridge that gap? If not, what makes this well-adjusted?

**Supplementary material 4**

*Topic guide for participants of the SELFIE intervention*

In black: Version 1 (November 2020)

In orange: Addition version 2 (March 2021)

In blue: Addition version 3 (April 2021)

- What do you consider the outcomes of the SELFIE intervention to have been for yourself (during and directly after the six weeks intervention period)?
- Can you give an example of [outcome named in the previous question]?
- Do you think that the outcomes would have been the same for different periods in your life? If not, in what ways have they been different due to the differing circumstances?
- Do you think that the outcomes would have been the same for friends/classmates/fellow students if they had received the SELFIE intervention?
- We are very curious about how the SELFIE intervention causes its outcomes. We identified different elements of the SELFIE intervention, namely the specific exercises, the use of a smartphone application in daily life, and it is guided self-help with supporting contact with a SELFIE therapist while receiving the SELFIE intervention.
  - How do you think **the specific exercises** have caused or helped to cause [outcome identified by respondent]? (if needed after an initial answer, prompt the different SELFIE intervention exercises)
  - How do you think the use of a smartphone application **in daily life** has caused or helped to cause [outcome identified by respondent]?
  - How do you think the **guidance by the SELFIE therapist** has caused or helped to cause [outcome identified by respondent]? (ask for possible differences between personal contact and email contact)
- Has the SELFIE intervention changed the way you think or feel about yourself in any way?
- Have you experienced difficulties when following the SELFIE intervention?
- There are lots of ideas about how the SELFIE intervention may actually work, and we think it probably works differently in different places or for different people. One of those ideas is [see following examples]. Has it worked at all like that here/for you? Can you give an example? And if not, what is your view on this?

- If participants experience the role of the SELFIE therapists as less important than their own attribution and active participation, then they will attribute the effect of the intervention increasingly to themselves.
- Repeatedly experiencing a short change in momentary self-esteem due to practicing the exercises often in daily life, helps to establish a change in general self-esteem.
- If participants experience the intervention on a smartphone application as anonymous and accessible, they will experience less shame to enter input on the app and feel more at ease working with the app.

The above question was replaced by the following question in the 3rd version of the topic guide:

- There are lots of ideas about how the SELFIE intervention actually works, and we think it probably works differently in different places or for different people.
    - Do you feel that your mood influenced how you perceived the beep questionnaire? If yes, how. If not, do you think your mood was of influence in any other way while receiving the SELFIE intervention? Or has it not been of influence at all?
    - Some participants mentioned that being asked how they feel, repeatedly during the day, made them experience one day as several smaller blocks of time (rather than experiencing the day as one long period, or a week as one long period). Is this something you recognize? If so, did it influence the way you participated in the SELFIE intervention?
    - Could you please describe, as accurately as possible, in what way the contact with the SELFIE therapist influenced your participation in the SELFIE intervention?

- What is it about the way the SELFIE intervention was offered that made a difference to how you did or didn't experience change?

- If you could change something about the SELFIE intervention to make it work more effectively for you, what would you change and why?

- What else do you think we need to know, to really understand how the SELFIE intervention has, or hasn't, worked well for you?

- Has the SELFIE intervention had long-term effects on you? If yes, what effect and what has caused that effect? If not, why do you think the outcome for you hasn't endured?

**Supplementary material 5**

*Topic guide for the focus group with SELFIE therapists on May 7th, 2021*

- What, to you, are the most important differences between participants that have been of influence on experiencing desired outcomes of the SELFIE intervention? And how can you understand the way it affected the outcome?

- What to you is of relevance for the SELFIE intervention to work successfully or not (for example the offering of the intervention through a smartphone application, guided self-help, online, number and content of exercises, the role of the SELFIE therapist)?

- What is your personal attitude towards guided self-help through a smartphone application?

- How do you perceive the role of the SELFIE therapist within this intervention?

- Have you been able in any way to practice techniques like modeling and shaping? If so, what effect did this have on the youngster do you think?

- There are lots of ideas about how the SELFIE intervention may actually work, and we think it probably works differently in different places or for different people. One of those ideas is [see following examples]. Is that something you recognize? Can you give an example? And if not, what is your view on this?
    - If participants experience the role of the SELFIE therapists as less important than their own attribution and active participation, then they will attribute the effect of the intervention increasingly to themselves.
    - By repeatedly experiencing a short change in momentary self-esteem due to practicing the exercises often in daily life, helps to establish a change in general self-esteem.
    - If participants experience the intervention on a smartphone application as anonymous and accessible, they will experience less shame to enter input on the app and feel more at ease working with the app.

- It seems that participants first experienced a cognitive change, and subsequently an affective change. Is that something you recognize, and if so, how? If not, how do you think a process of change is formed?

- Repetition and the offering of the SELFIE intervention in daily life, seem to support the youngster to practice specific elements of cognitive change in a more automated manner, how does that work in your opinion?

**Supplementary Material 6**

In the process of developing an IPT, the data led to formulating the aspired outcome of the SELFIE intervention as follows: "A measurably improved self-image characterized by an attention switch towards positive qualities of oneself, a positive perception of general well-being, more resilience, and behavior that fits a more positive self-image." Furthermore, the development of the initial programme theory (IPT) was structured by the programme architecture of the SELFIE intervention. One pillar of this programme is that it is an ecological momentary intervention (EMI). The development of IPTs regarding this pillar is described below.

<u>IPT 1</u>

An EMI offered as an application on a smartphone is thought to be easily accessible, person-tailored (through personalized feedback or personal goals) and furthermore offers anonymity in accessing the intervention. The aspect of anonymity in relation to self-esteem is thought to be linked to decreased experience of shame, as illustrated by the following quote:

> "*[…] the filling in of exercises in the application was less shameful to her, so she managed better to formulate positive thoughts about herself without experiencing shame reactive to interpersonal contact because nobody reacted to it at that moment, she did that by herself. […]*' FG_SH (346)

Perhaps, due to limited feelings of shame, anonymity may be supportive of engagement in the intervention and consequently outcome, as mentioned in the stakeholders focus group:

> "*[…] that practising in anonymity supports the ease of practising, and therefore leads to more positive experiences*." FG_SH (378)

The following IPT takes into account the above described characteristics of and EMI targeting self-esteem: '**If participants experience the intervention as personalized, anonymous, and easily accessible, then they will be more comfortable with their input and can participate in the SELFIE intervention without feelings of shame.**'

IPT 2

The development of the SELFIE intervention is rooted in ecological psychology and for EMI it is hypothesized that changing experiences is best done in the context. Therefore, a stakeholder proposes that the SELFIE intervention shapes self-esteem in the context, and furthermore, that the impact on self-esteem is therefore stronger and consequently may lead to sustained change in self-esteem. The repetitive nature of this exposure is thought to be effective in generating an outcome.

> "*[…] without having to talk to someone, a person, just on your own […] at repeated moments when you are "nudged" in a certain mindset, I think that does have an effect*." FG_SH (454)

Generalization, that fits within the CBT framework to bring therapeutic interventions into a clients natural environment, seems to be an important aspect in facilitating this change (Heron & Smyth, 2010; Myin-Germeys, Birchwood, & Kwapil, 2011). An IPT encompasses the aforementioned elements as follows: '**If a change in momentary self-esteem is established repeatedly and under different circumstances through the use of a smartphone application, then this will support the generalizability of this effect and support change in general self-esteem**.'

IPT 3

Due to the inherent use of (mobile) technology, EMI's may be subject to technical malfunctions. Literature points towards technical malfunction being an influential factor in engagement and adherence to a programme (Garrido et al., 2019). During the implementation phase of the SELFIE intervention, stakeholders had indeed experienced technical problems with for example logging into the smartphone application and, in a few instances experienced irritation or decreased motivation in study participants. Furthermore, the use of reminder beeps may be perceived as annoying by participants in the context of a digital age with many (social media) notifications, as illustrated by the following quote:

> "*They [youth] receive 500 beeps a day*" EI_1 (464-470).

Therefore, an IPT on the technical aspects of the SELFIE intervention was developed: '**If a technical malfunction is present or the reminder beeps are being perceived negatively, then the effect of the intervention will be limited and drop-out may occur.**'
